# Supplementary material for: LEADeR role of miR-205 host gene as long noncoding RNA in prostate basal cell differentiation
Source: Nat Commun. 2019 Jan 18;10:307. doi: 10.1038/s41467-018-08153-2 (PMC6338800; doi:10.1038/s41467-018-08153-2)
Supplement: Supplementary file 1 — Supplementary Information [file 41467_2018_8153_MOESM1_ESM.pdf]

SUPPLEMENTARY INFORMATION

***LEADeR* role of *miR-205* host gene as long non-coding RNA in prostate basal cell differentiation**

Profumo, Forte, Percio, Rotundo et al.

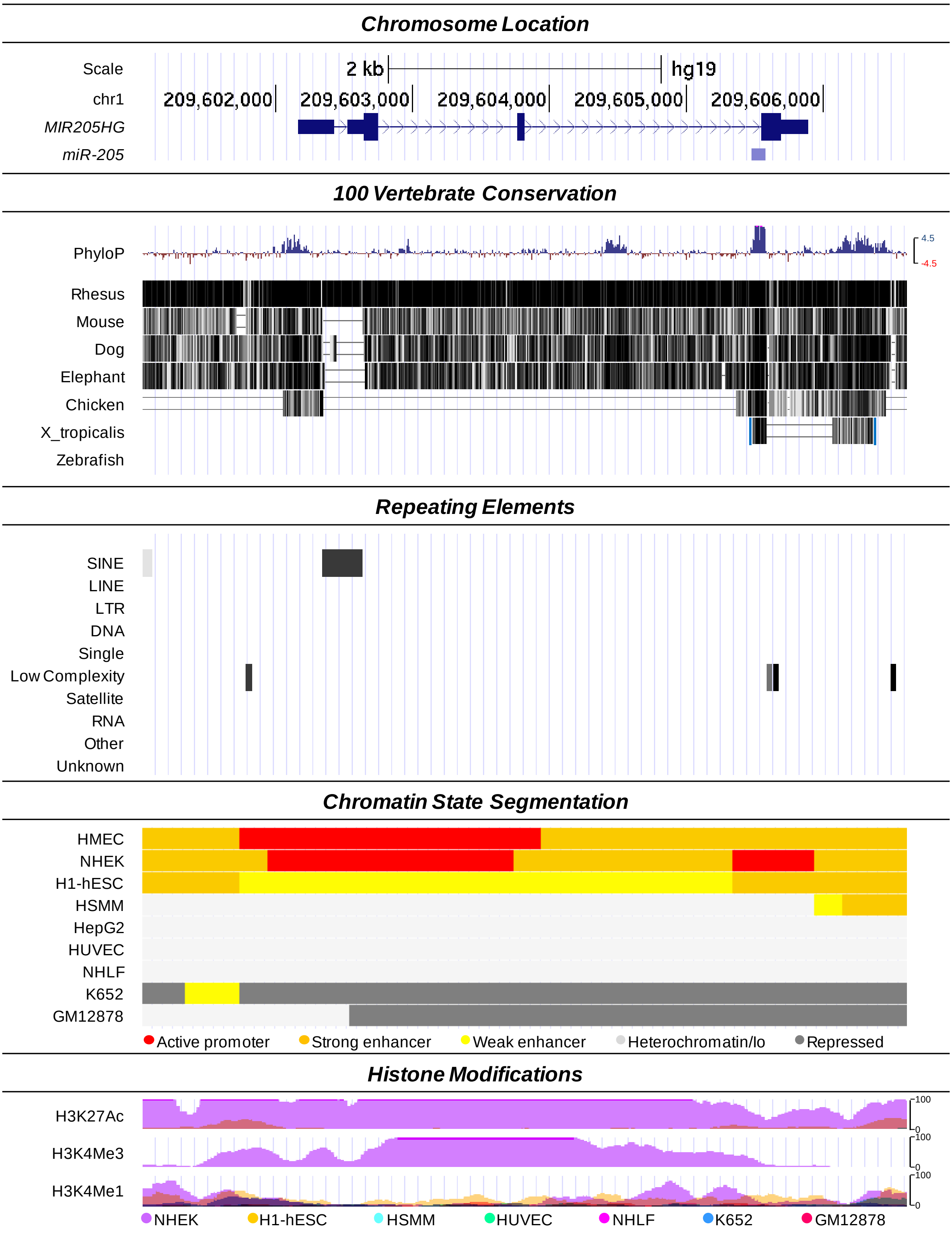

Supplementary Figure 1. ***LEADR/MIR205HG* locus.** *MIR205HG* locus as from UCSC Genome Browser (Human GRCh37/hg19 Assembly). From top to bottom, *i)* chromosome location of *LEADR/MIR205HG* NCBI RefSeq and *miR-205* precursor, *ii)* sequence conservation across 100 vertebrate species (shown as PhyloP and Multiz Alignments), *iii)* repeating elements (with evident SINE/*Alu* element spanning *LEADR* exon-1 and 2), *iv)* chromatin state segmentation and *v)* histone modifications in ENCODE cell lines. Color codes for chromatin state segmentation and for

ENCODE cell lines analyzed for histone modifications (namely HMEC, Human Mammary Epithelial Cells; NHEK, Normal Human Epidermal Keratinocytes; H1-hESC, Human Embryonic Stem Cells; K562, chronic myeloid leukemia cell line; GM12878, lymphoblastoid cell line; HSMM, Human Skeletal Muscle Myoblasts; HUVEC, Human Umbilical Vein Endothelial Cells; NHLF, Normal Human Lung Fibroblasts) are reported below each panel. Tracks for histone modifications are shown in packed form. y-scale shown on the right side, when applicable.

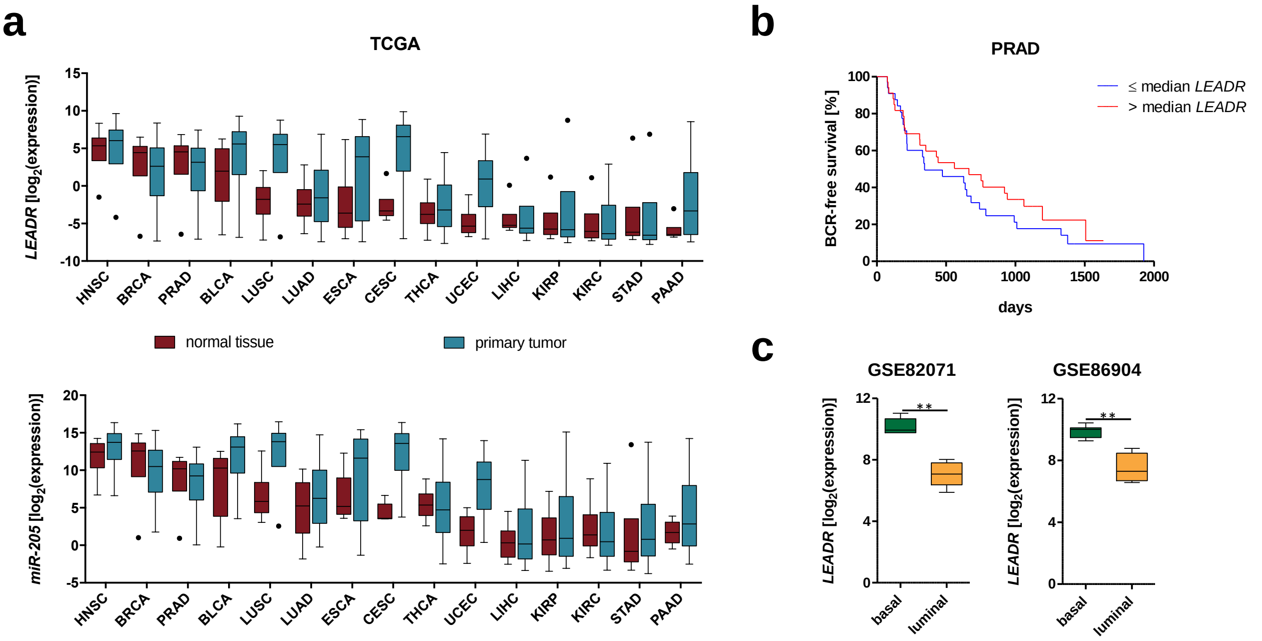

Supplementary Figure 2. ***LEADR/MIR205HG* expression in human tumors and prostate cells.** (**a**) Tukey's box plots of *LEADR/MIR205HG* (*top*) and *miR-205* (*bottom*) expression levels in different human tumors compared to healthy counterparts, as from TCGA. Only cancer types with at least 100 tumor and 3 normal samples with available *MIR205HG* and *miR-205* expression data were considered for the analysis. [BLCA (Bladder Urothelial Carcinoma), BRCA (Breast Invasive Carcinoma), CESC (Cervical Squamous Cell Carcinoma and Endocervical Adenocarcinoma), ESCA (Esophageal Carcinoma), HNSC (Head and Neck Squamous Cell Carcinoma), KIRC (Kidney Renal Clear Cell Carcinoma), KIRP (Kidney Renal Papillary Cell Carcinoma), LIHC (Liver Hepatocellular Carcinoma), LUAD (Lung Adenocarcinoma), LUSC (Lung Squamous Cell Carcinoma), PAAD (Pancreatic Adenocarcinoma), PRAD (Prostate Adenocarcinoma), STAD (Stomach Adenocarcinoma), THCA (Thyroid Carcinoma), UCEC (Uterine Corpus Endometrial Carcinoma)]. (**b**) Kaplan-Meier curve reporting biochemical relapse (BCR)-free survival of patients with *LEADR* expression lower (blue) or higher (red) than the median value from TCGA-PRAD. Neither log-rank nor Wilcoxon tests showed significance differences in relapse occurrence. (**c**) Tukey's box plots of *LEADR/MIR205HG* expression in basal/luminal cell subpopulations from two publicly available gene expression datasets of cells isolated from human normal prostate. **p<0.01 (Student's t-test). Source data are provided as a Source Data file, together with n of all experiments.

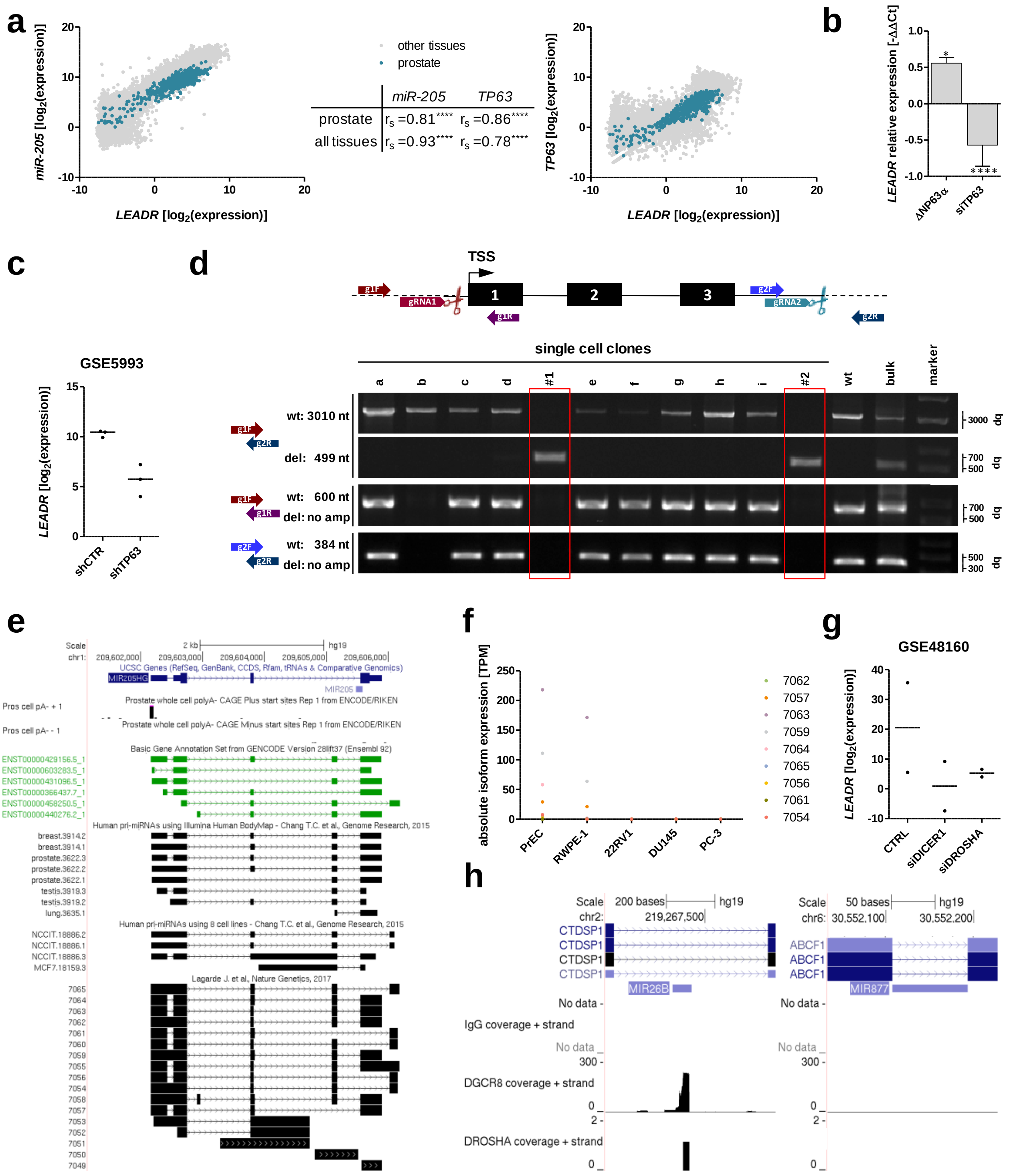

	miR-205	*TP63*
prostate	r_s =0.81****	r_s =0.86****
all tissues	r_s =0.93****	r_s =0.78****

Supplementary Figure 3. *LEADR* expression is regulated by p63 and *miR-205* biogenesis is Drosha- and Dicer-dependent. (**a**) Scatter plot of *LEADR* and *miR-205* (*left*), or *TP63* (*right*) expression in TCGA. Spearman correlation coefficients (r_s) and *p*-values are reported in the embedded table. (**b**) qRT-PCR showing modulation of *LEADR* expression levels upon over-expression (ΔNp63α) or silencing (siTP63) of p63 in RWPE-1 cells, as compared to relative controls (EV or siCTR, respectively). Mean + s.d. (n=2 and 8, respectively) plotted. (**c**) Dot plot of *LEADR/MIR205HG* reduction upon p63 silencing by shRNA in cervical carcinoma cells (GSE5993). Each sample shown. Line at median. (**d**) Genotyping of CRISPRed RWPE-1 cells. Location of gRNAs used for genomic deletion of *LEADR* and of primers used for genotyping PCR is shown. Both bulk population and single cell clones (from a to i) are reported. Clones

characterized by homozygous *LEADR* deletion are indicated as clone #1 and clone #2. (**e**) *LEADR/MIR205HG* transcript structures as from: RefSeq; Genecode v28lift37 Basic; genome-wide high-resolution remapping of pri-miRNAs performed by Chang T.C. *et al.*, Genome Research, 2015 (SRP057660); targeted RNA capture with third-generation long-read sequencing technology performed by Lagarde J. *et al.*, Nature Genetics, 2017 (GSE93848). Results of CAGE experiments on prostate cells from ENCODE/RIKEN showing *LEADR* putative TSS on the positive strand are reported. (**f**) Absolute expression of *LEADR* transcript isoforms in normal (PrEC and RWPE-1) and tumor cell lines (22RV1, DU145 and PC-3) as from RNA-Seq data (GSE75035 and GSE25183). (**g**) Dot plot of *miR-205* expression upon silencing of *DROSHA* or *DICER1*. Each sample shown. Line at median. (**h**) RNA-Seq peaks of DROSHA and DGCR8 as from CLIP experiments (GSE61979) shown for *miR-26b* (*left*) and *miR-877* (*right*). IgG peaks reported for comparison. $^{*}p<0.05$; $^{****}p<0.0001$ (Student's t-test). Source data are provided as a Source Data file, together with n of all experiments.

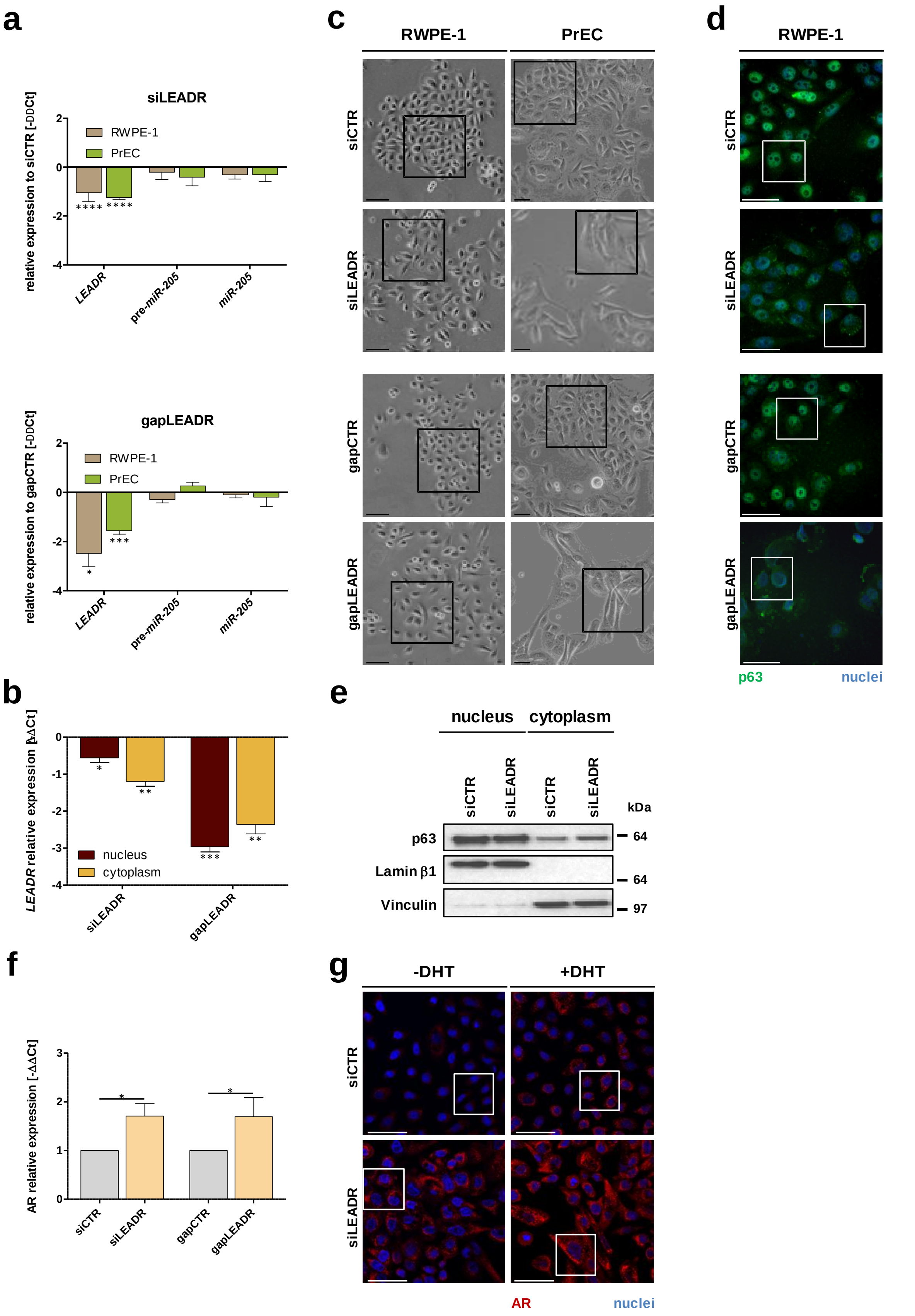

Supplementary Figure 4. *LEADR* regulates basal-luminal differentiation. (**a**) qRT-PCR showing *LEADR*, precursor and mature *miR-205* expression levels in immortalized (RWPE-1) or primary (PrEC) prostate basal cells upon *LEADR* silencing by si*LEADR* (*top*) or gapLEADR (*bottom*). Mean + s.d. plotted. (**b**) qRT-PCR showing the efficacy of siLEADR or gapLEADR in

abrogating the nuclear and cytoplasmic *LEADR* pools in RWPE-1 cells. Mean + s.d. (n=3 qRT-PCR measurements for each experimental condition) plotted, as compared to the specific control oligomers. (**c**) Full-size bright-field images showing morphological changes occurring in RWPE-1 (*left*) and PrEC (*right*) cells (day 3) upon *LEADR* silencing by siLEADR (*top*) or gapLEADR (*bottom*). Scale bar, 50 µm. Magnifications shown in main Fig. 5a. (**d**) Full-size immunofluorescence images showing cytoplasmic re-localization of p63 (green) upon *LEADR* silencing in RWPE-1 cells by siLEADR (*top*) or gapLEADR (*bottom*). Nuclei counterstained with DAPI (blue). Scale bar, 50 µm. Magnifications shown in main Fig. 5d. (**e**) Western blot showing cytoplasmic re-localization of p63 upon *LEADR* silencing in RWPE-1 cells by siLEADR. Lamin-β1 and Vinculin used as nuclear and cytoplasmic reference, respectively. (**f**) qRT-PCR showing changes in androgen receptor (AR) expression in PrEC cells upon *LEADR* silencing by siLEADR or gapLEADR. Mean + s.d. (n=3) plotted. (**g**) Full-size immunofluorescence images showing AR (red) expression in PrEC cells upon *LEADR* silencing by siLEADR, in the presence or absence of simultaneous stimulation with DHT. Nuclei counterstained with DAPI (blue). Scale bar, 50 µm. Magnifications shown in main Fig. 5f. $^{*}p<0.05$; $^{**}p<0.01$; $^{***}p<0.001$; $^{****}p<0.0001$ (Student's t-test). Source data are provided as a Source Data file, together with n of all experiments.

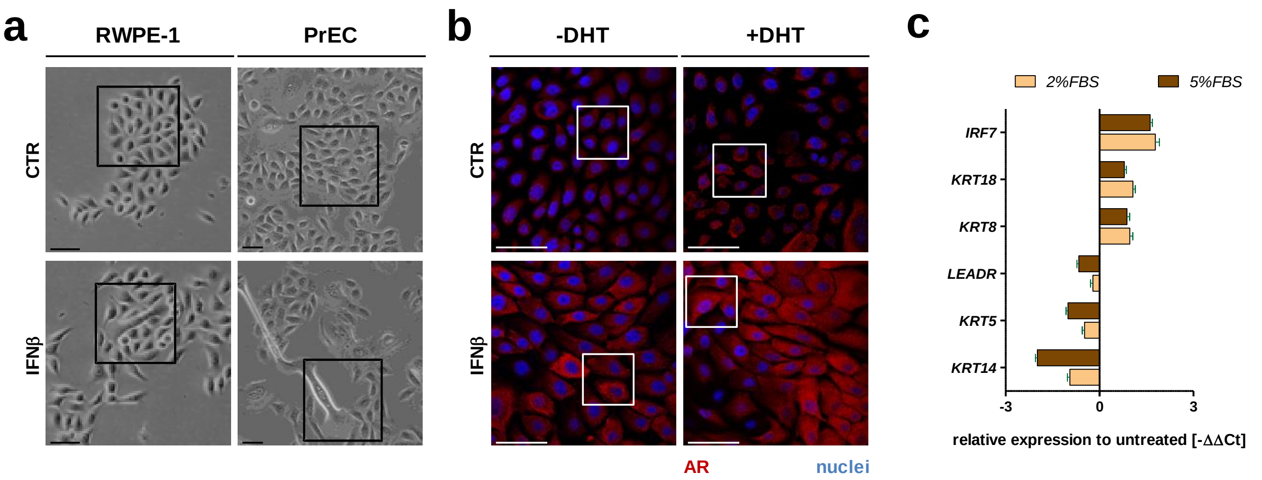

Supplementary Figure 5. Interferon induces basal-luminal differentiation. (**a**) Full-size bright-field images showing morphological changes occurring in RWPE-1 and PrEC cells (day 3) upon interferon-β1 treatment. Scale bar, 50 µm. Magnifications shown in main Fig. 6e. (**b**) Full-size immunofluorescence images showing AR (red) expression in PrEC cells upon interferon-β1 treatment in the presence or absence of simultaneous stimulation with DHT. Nuclei counterstained with DAPI (blue). Scale bar, 50 µm. Magnifications shown in main Fig. 6h. (**c**) qRT-PCR showing changes in basal/luminal cytokeratins, *IRF7*, and *LEADR* in PrEC cells upon serum stimulation. Mean + s.d. (n=3 qRT-PCR measurements for each experimental condition) plotted. Source data are provided as a Source Data file.

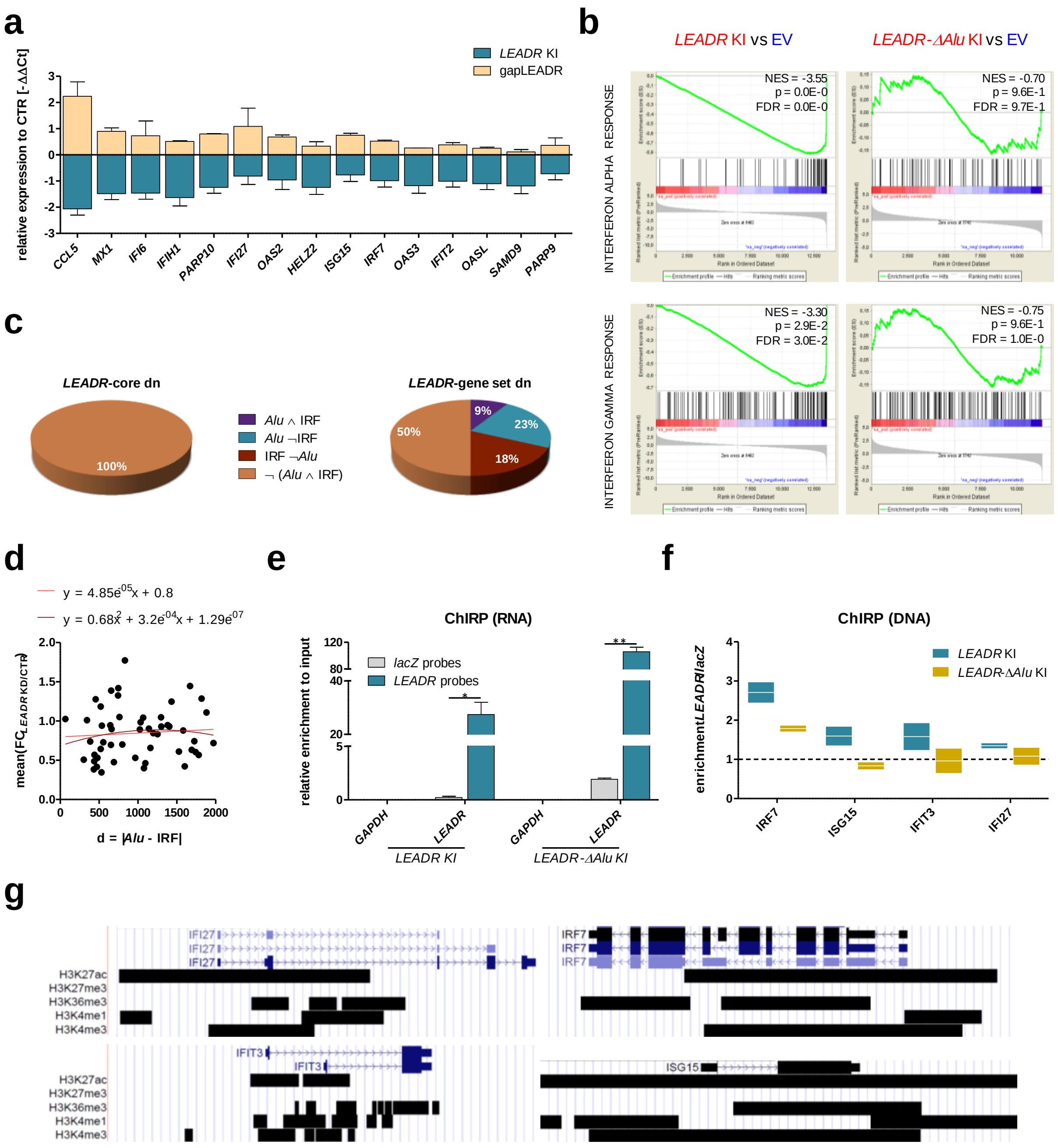

Supplementary Figure 6. Analysis of *LEADR* target genes. (**a**) qRT-PCR analysis showing the expression of genes randomly selected from '*LEADR*-core up' gene set upon *LEADR* silencing in RWPE-1 cells (gapLEADR) or overexpression (RefSeq) in DU145 cells. Mean + s.d. (n=2 and 3, respectively) plotted. (**b**) GSEA of interferon-related gene sets in DU145 knocked-in (KI) for the wild type (*LEADR*) or the *Alu*-deleted (*LEADR*-Δ*Alu*) form of *LEADR* RefSeq transcript. NES, p, and FDR values are indicated in each plot. (**c**) Pie charts of the frequency of *Alu*/IRF sites in '*LEADR*-core dn' and '*LEADR*-gene set dn'signatures. (**d**) Scatter plot showing lack of linear or polynomial correlation between intensity of regulation by *LEADR* (plotted as mean value of FCs of the two *LEADR* silencing experiments) and the relative distance between *Alu* and IRF-binding sites on target gene promoters. (**e**) qRT-PCR showing RNA retrieval upon ChIRP performed in *LEADR* or *LEADR*-Δ*Alu* knocked-in DU145 cells, using probes complementary to *LEADR* or to *lacZ*. Mean + s.d. (n=3 qRT-PCR measurements for each experimental condition) plotted. (**f**) ChIRP-qPCR detection of *LEADR* occupancy on representative target loci (normalized to unrelated genomic region) in *LEADR* or *LEADR*-Δ*Alu* knocked-in DU145 cells. Line at mean plotted (n=2). (**g**)

Histone modifications found in promoters of selected '*LEADR*-signature' genes, as from GSE63094 ChIP-Seq data on RWPE-1 cells, viewed on UCSC Genome Browser. $^{*}p<0.05$; $^{**}p<0.01$ (Student's t-test). Source data are provided as a Source Data file, together with n of all experiments.

Fig. 5c

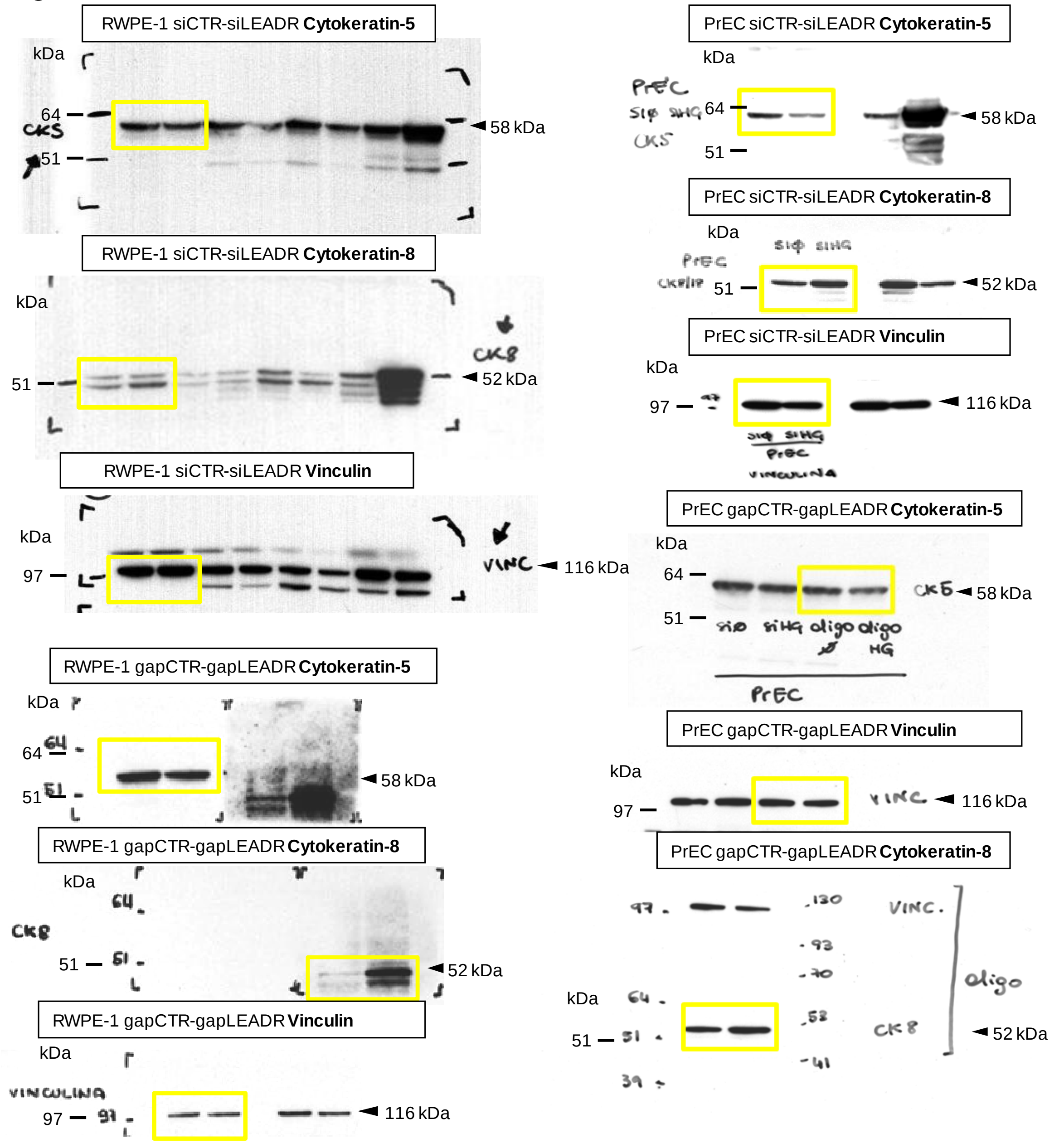
RWPE-1 siCTR-siLEADR Cytokeratin-5
kDa
64
51
58 kDa
RWPE-1 siCTR-siLEADR Cytokeratin-8
kDa
51
52 kDa
RWPE-1 siCTR-siLEADR Vinculin
kDa
97
116 kDa
RWPE-1 gapCTR-gapLEADR Cytokeratin-5
kDa
64
51
58 kDa
RWPE-1 gapCTR-gapLEADR Cytokeratin-8
kDa
51
52 kDa
RWPE-1 gapCTR-gapLEADR Vinculin
kDa
97
116 kDa
PrEC siCTR-siLEADR Cytokeratin-5
kDa
64
51
58 kDa
PrEC siCTR-siLEADR Cytokeratin-8
kDa
51
52 kDa
PrEC siCTR-siLEADR Vinculin
kDa
97
116 kDa
PrEC gapCTR-gapLEADR Cytokeratin-5
kDa
64
51
58 kDa
PrEC gapCTR-gapLEADR Vinculin
kDa
97
116 kDa
PrEC gapCTR-gapLEADR Cytokeratin-8
kDa
51
52 kDa

Fig. 5e

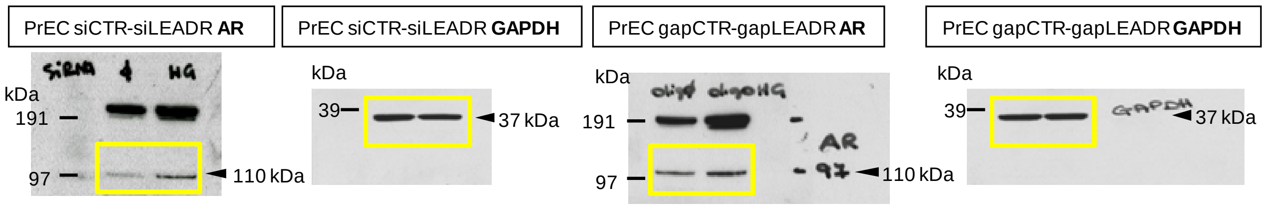
PrEC siCTR-siLEADR AR
kDa
191
97
110 kDa
PrEC siCTR-siLEADR GAPDH
kDa
37 kDa
PrEC gapCTR-gapLEADR AR
kDa
97
110 kDa
PrEC gapCTR-gapLEADR GAPDH
kDa
37 kDa

Fig. 6g

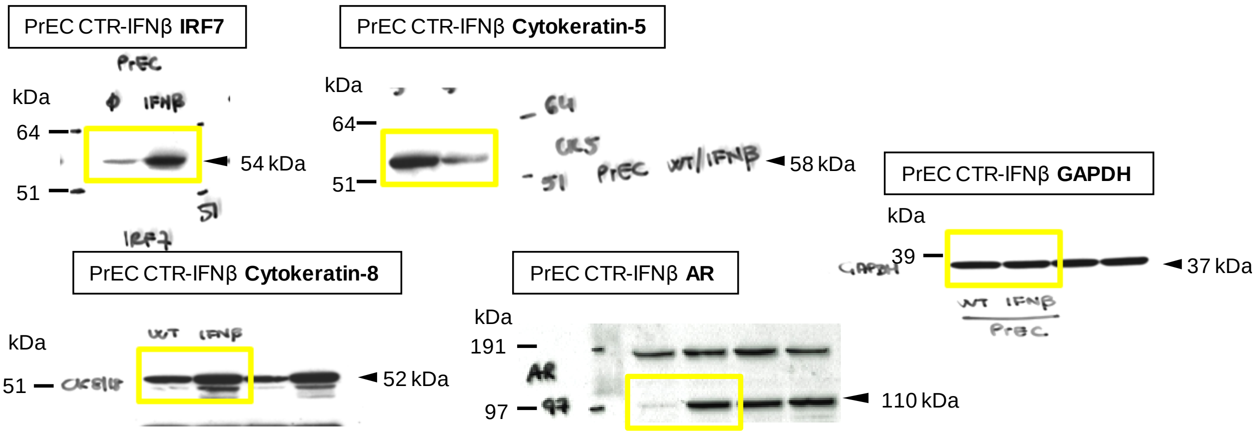

Fig. 6j

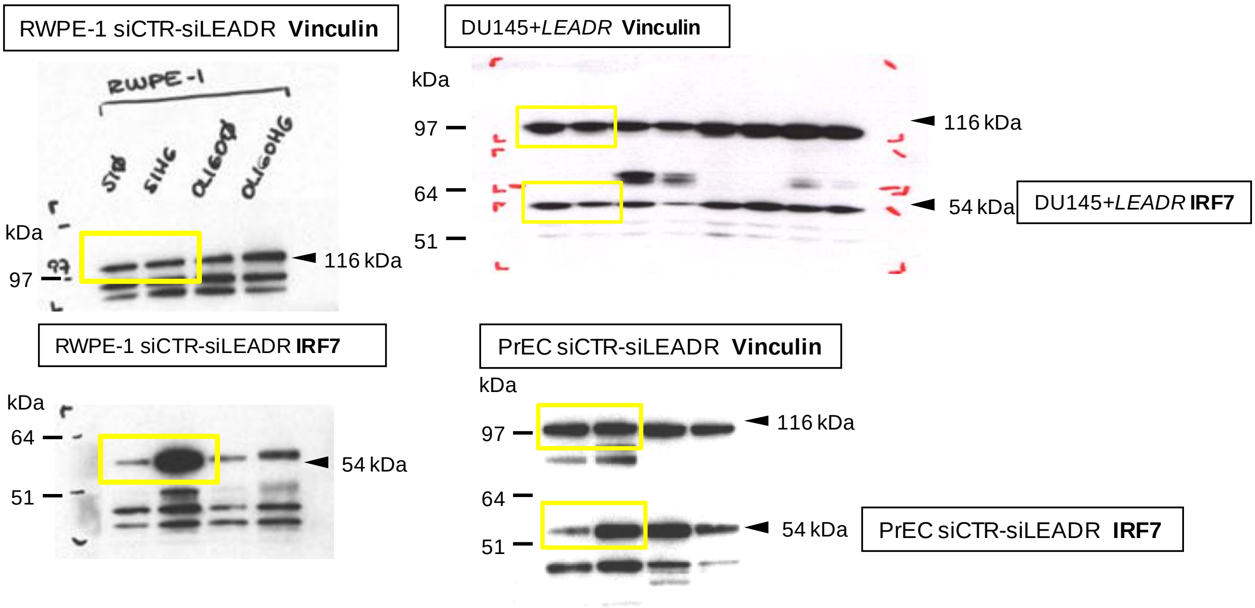

Fig. 9c

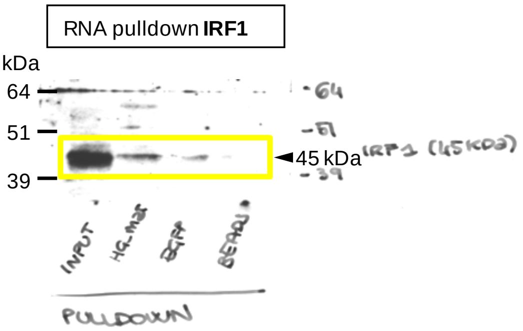

Supplementary Fig. 4e

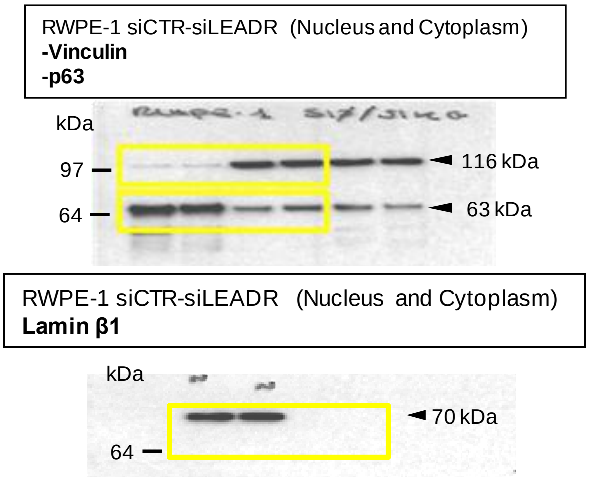

Supplementary Figure 7. Uncropped blots. Uncropped images of all blots shown in main and supplementary figures. Molecular weight markers are indicated on the left side of each blot, whereas predicted molecular weight of the protein of interest indicated by an arrow on the right.

**Supplementary Table 1. CPAT analysis on *bona fide LEADR/MIR205HG* transcripts.**						
transcript	RNA size	ORF size	Ficket score	hexamer score	coding probability	coding label
7062	1074	213	0.537	-0.07616	0.009241	no
7057	918	333	1.0013	0.032806	0.233312	no
7063	983	213	0.537	-0.07616	0.009292	no
7059	1096	237	0.5842	-0.07618	0.013809	no
7064	1007	237	0.5842	-0.07618	0.013883	no
7065	912	237	0.5842	-0.07618	0.013962	no
7056	757	213	0.537	-0.07616	0.009419	no
7061	703	312	0.4714	-0.18602	0.010842	no
7054	851	213	0.537	-0.07616	0.009366	no

Supplementary Table 2. Sequences of siRNAs and gapmers

siRNAs	Sequence 5’-3’
siCTR	5’-GCAUACAAUGGAGUUGUUA-3’
siLEADR	5’-AACUGGGUGCUUUAUAUAGGA-3’
siTP63	5’-ACAAUUUCAUGUGUAACAGCA-3’
siDROSHA1	5’-AACGAGUAGGCUUCGUGACUU-3’
siDROSHA2	5’-AAGGACCAAGUAUUCAGCAAG-3’

gapmers	Sequence 5’-3’
gapCTR	5'-TTAACAACTCCATTGTATGC-3'
Intronic gapmers	
gapINT1	5’-GAATATGGCATTTTCCAGTC-3’
gapINT2	5’-CAGTCTGTTGGCTGTAATAA-3’
Exon 2-3 junction gapmer	
gapLEADR	5'-ATGACAGTGTCCTATATAAAGC -3'

Supplementary Table 3. Guide RNAs and primers used for CRISPR/Cas9 editing

Guide RNAs used for CRISPR/Cas9 editing

**Guide_RNA_1 (g1)**	5'-CGATTAGGTAGGTCTCTGGG-3'
**Guide_RNA_2 (g2)**	5'-GCCCAGAAGGCTAGATGCGT-3'

Primers used for genotyping

		Expected amplicon
g1-F	5'-CAAGGGGAGCAGCAGACTTA-3'	wt: 3010 bp - del 499 bp
g2-R	5'-GAAATGCCCATCAACCCTTT-3'	
g1-F	5'-CAAGGGGAGCAGCAGACTTA-3'	wt: 600 bp - del: no amplification
g1-R	5'-GGTGAGCAAGAGGGACTCAG-3'	
g2-F	5'-GATCTCTGCCTGCCAACAAG-3'	wt: 384 bp - del : no amplification
g2-R	5'-GAAATGCCCATCAACCCTTT-3'	

Supplementary Table 4. TaqMan Assays	
**Gene**	**Assay**
MIR205HG*	*Hs03405498_m1*
**pre-*miR-205***	*Hs04231469_s1*
KRT5	*Hs00361185_m1*
KRT14	*Hs00265033_m1*
KRT8	*Hs01595539_m1*
KRT18	*Hs02827483_g1*
IRF7	*Hs01014809_g1*
DROSHA	*Hs00203008_m1*
MALAT1	*Hs00273907_s1*
SNORA74A	*Hs03298571_s1*
GAPDH	*PN4326317E*
CCL5	*Hs00982282_m1*
MX1	*Hs00895608_m1*
IFI6	*Hs00242571_m1*
IFIH1	*Hs00223420_m1*
PARP10	*Hs00361105_m1*
IFI27	*Hs01086373_g1*
OAS2	*Hs00942643_m1*
HELZ2	*Hs00375688_m1*
ISG15	*Hs01921425_s1*
OAS3	*Hs00196324_m1*
IFIT2	*Hs01922738_s1*
OASL	*Hs00984387_m1*
SAMD9	*Hs00539471_s1*
PARP9	*Hs00967084_m1*
AR	*Hs00907244_m1*

**microRNA**	**Assay**
hsa-miR-205	TaqMan microRNA assay *000509*
hsa-miR-200b	TaqMan microRNA assay *002274*
hsa-miR-200c	TaqMan microRNA assay *2300*
hsa-miR-26b	TaqMan microRNA assay *000407*
hsa-miR-877	TaqMan microRNA assay 241029
RNU48	TaqMan microRNA assay *001006*

*alias *LEADR*: assay covering exon2-exon3 boundary.

Supplementary Table 5. Primers for end-point RT-PCR and for Sybr green qPCR

Primers for end-point RT-PCR

Gene	Forward	Reverse
miR-205 byproduct	5'-CGAGCTCAGTTATGGCACAC-3'	5'-GGGAGTCTAAGGGCAGCAG-3'
ACTIN	5'-CAACGGCTCCGGCATGTG-3'	5'-CTCCTTAATGTCACGCACGA-3'

Primers for Sybr green qPCR (used for ChIP and ChIRP)

Gene	Forward	Reverse
IFI27	5'-GTGGCCCATGTGATAAGCTG-3'	5'-CAAGGCTTTAATGGGAGGGC-3'
IFIT3	5'-CTGATGCGTGCCCTACTCT-3'	5'-ATGACTGCCCTCTGTGTCTC-3'
IRF7	5'-AGTAGGGAGGAGTGGAGGG-3'	5'-GTCCACCTCCCATTACCCAC-3'
ISG15	5'-GAGGCTGAGGTG AGA GGA TC-3'	5'-GGT CTC TCA CTC TGT CGC C-3'
ISG20	5'-CTTCCAGCATCAGTAGTGGC-3'	5'-ACTACCCATCCCCGCTTCAG-3'
CCL5	5'-CACAAGAGGAAACCAAGGCC-3'	5'-GAAGTGGGAGTGAGGGCAA-3'
OAS2	5'-CATGTGATTGTGGAGGCTGG-3'	5'-CAAGGAAGAGGGAATTCGGC-3'
OAS3	5'-GTTGGGAGGAGTCAGTGAGAG-3'	5'-GAGAAGTCCTACCTCGCTGTC-3'
**control region *12700F***	5'-GCCTGAGTTACCACACCCAGT-3'	5'-TCAGCCACTGTGAAAAGCAG-3'

Supplementary Table 6. Probes used in ChIRP experiments	
**Probe name**	**Sequence**
**LEADR1**	5’-GGTGAGCAAGAGGGACTCAG-3’
**LEADR2**	5’-AAACTGGTTTTTCCAAGTCA-3’
**LEADR3**	5’-GTATGGTTGAGAATTGAGCT-3’
**LEADR4**	5’-CGGTCCTGAATAGTTGATTT-3’
**LEADR5**	5’-ACTCCTGAGGAAAGGTGGGG-3’
**LEADR6**	5’-AGGCCTGTGCGGAACAGAAA-3’
**LEADR7**	5’-CCTATATAAAGCACCCAGTT-3’
**LEADR8**	5’-CTCCAAGATGGGTACTTGAG-3’
**LEADR9**	5’-ACGCAAATTTTCATAGCATC-3’
**LEADR10**	5’-TTACAAGTTACAGAAAACGC-3’
**LacZ1**	5’-CCAGTGAATCCGTAATCATG-3’
**LacZ2**	5’-TCACGACGTTGTAAAACGAC-3’
**LacZ3**	5’-GCTGATTTGTGTAGTCGGTT-3’
**LacZ4**	5’-TTAAAGCGAGTGGCAAGATG-3’
**LacZ5**	5’-AGACGATTCATTGGCACCAT-3’
**LacZ6**	5’-TGATCACACTCGGGTGATTA-3’
**LacZ7**	5’-TTTACCTTGTGGAGCGACAT-3’
**LacZ8**	5’-GTTCAGGCAGTTCAATCAAC-3’
**LacZ9**	5’-AGTTTTCTTGCGGCCCTAAT-3’
**LacZ10**	5’-ATGTCTGACAATGGCAGATC-3’

Supplementary Table 7. Site directed mutagenesis primers	
**Sequence**	
**ΔALU_SENSE**	5'-CTCACTGCAGCCTCAACCTCCCAGACCGCATCCGGCCTCATGTTC-3'
**ΔALU_ANTISENSE**	5'-GAACATGAGGCCGGATGCGGTCTGGGAGGTTGAGGCTGCAGTGAG-3'
