# Supplementary material for: LEADeR role of miR-205 host gene as long noncoding RNA in prostate basal cell differentiation
Source: Nat Commun. 2019 Jan 18;10:307. doi: 10.1038/s41467-018-08153-2 (PMC6338800; doi:10.1038/s41467-018-08153-2)
Supplement: Supplementary file 3 — Description of Additional Supplementary Files [file 41467_2018_8153_MOESM3_ESM.docx]

**Description of Additional Supplementary Files**

File Name: Supplementary Data 1

Description: LEADR-related gene sets and signatures

File Name: Supplementary Data 2

Description: FIMO analysis of de novo motifs on LEADR transcript

File Name: Supplementary Data 3

Description: PSCAN analysis on 'LEADR-core up' genes

File Name: Supplementary Data 4

Description: Antibodies used in this study

File Name: Supplementary Data 5

Description: Description of publicly available datasets used in this study

File Name: Supplementary Data 6

Description: Customly defined basal and luminal specific gene sets from publicly available data
