# Supplementary material for: LEADeR role of miR-205 host gene as long noncoding RNA in prostate basal cell differentiation
Source: Nat Commun. 2019 Jan 18;10:307. doi: 10.1038/s41467-018-08153-2 (PMC6338800; doi:10.1038/s41467-018-08153-2)
Supplement: Supplementary file 11 — Reporting Summary [file 41467_2018_8153_MOESM11_ESM.pdf]

nature research

Corresponding author(s): Paolo Gandellini

Reporting Summary

Nature Research wishes to improve the reproducibility of the work that we publish. This form provides structure for consistency and transparency in reporting. For further information on Nature Research policies, see Authors & Referees and the Editorial Policy Checklist.

Statistical parameters

When statistical analyses are reported, confirm that the following items are present in the relevant location (e.g. figure legend, table legend, main text, or Methods section).

n/a	Confirmed	
☐	☒	The exact sample size (*n*) for each experimental group/condition, given as a discrete number and unit of measurement
☐	☒	An indication of whether measurements were taken from distinct samples or whether the same sample was measured repeatedly
☐	☒	The statistical test(s) used AND whether they are one- or two-sided<br>*Only common tests should be described solely by name; describe more complex techniques in the Methods section.*
☒	☐	A description of all covariates tested
☐	☒	A description of any assumptions or corrections, such as tests of normality and adjustment for multiple comparisons
☐	☒	A full description of the statistics including central tendency (e.g. means) or other basic estimates (e.g. regression coefficient) AND variation (e.g. standard deviation) or associated estimates of uncertainty (e.g. confidence intervals)
☒	☐	For null hypothesis testing, the test statistic (e.g. *F*, *t*, *r*) with confidence intervals, effect sizes, degrees of freedom and *P* value noted<br>*Give P values as exact values whenever suitable.*
☒	☐	For Bayesian analysis, information on the choice of priors and Markov chain Monte Carlo settings
☒	☐	For hierarchical and complex designs, identification of the appropriate level for tests and full reporting of outcomes
☒	☐	Estimates of effect sizes (e.g. Cohen's *d*, Pearson's *r*), indicating how they were calculated
☐	☒	Clearly defined error bars<br>*State explicitly what error bars represent (e.g. SD, SE, CI)*

Our web collection on statistics for biologists may be useful.

Software and code

Policy information about availability of computer code

Data collection	No software was used.
Data analysis	Data were analyzed using R 3.4 and GraphPad Prism 5.

For manuscripts utilizing custom algorithms or software that are central to the research but not yet described in published literature, software must be made available to editors/reviewers upon request. We strongly encourage code deposition in a community repository (e.g. GitHub). See the Nature Research guidelines for submitting code & software for further information.

Data

Policy information about availability of data

All manuscripts must include a data availability statement. This statement should provide the following information, where applicable:

- Accession codes, unique identifiers, or web links for publicly available datasets
- A list of figures that have associated raw data
- A description of any restrictions on data availability

I confirm that the data availability statement is included in the manuscript and copied here below.
"Transcriptomic data from prostate cells modulated for MIR205HG/LEADR expression generated in our laboratory were deposited at Gene Expression Omnibus, with accession number GSE104003 [https://www.ncbi.nlm.nih.gov/geo/query/acc.cgi?acc=GSE104003]. Accession codes (and web links) for publicly available

datasets have been provided in the Methods section and in Supplementary Data 5. The source data underlying Figs 1a-c, 1e-h, 2b-f, 3a-b, 4b-c, 5b-c, 5e, 5g, 5i, 6f-g, 6i-j, 7d, 8d-e, 9a-e, and Supplementary Figs 2a, 2c, 3b-c, 3f-g, 4a-b, 4e-f, 5c, 6a, and 6e-f are provided as a Source Data file. All relevant data are available from the authors."

Field-specific reporting

Please select the best fit for your research. If you are not sure, read the appropriate sections before making your selection.

☒ Life sciences ☐ Behavioural & social sciences ☐ Ecological, evolutionary & environmental sciences

For a reference copy of the document with all sections, see nature.com/authors/policies/ReportingSummary-flat.pdf

Life sciences study design

All studies must disclose on these points even when the disclosure is negative.

Sample size	No sample size estimation was required for our experiments.
Data exclusions	No data were excluded.
Replication	Replicates were measured in terms of mean and standard deviation (sd); sample sizes were reported in the legends and in the Source Data File
Randomization	Our experiments do not need randomization.
Blinding	Our experiments do not need blinding.

Reporting for specific materials, systems and methods

Materials & experimental systems

n/a	Involved in the study
☒	☐ Unique biological materials
☐	☒ Antibodies
☐	☒ Eukaryotic cell lines
☒	☐ Palaeontology
☒	☐ Animals and other organisms
☒	☐ Human research participants

Methods

n/a	Involved in the study
☒	☐ ChIP-seq
☒	☐ Flow cytometry
☒	☐ MRI-based neuroimaging

Antibodies

Antibodies used	All this information is included in Supplementary Data 4.
Validation	All this information is included in Supplementary Data 4.

Eukaryotic cell lines

Policy information about cell lines

Cell line source(s)	Information on cell line source is provided in the section 'Cell lines and cell-based experiments' in the Methods: "Established human cell lines were purchased from American Type Culture Collection (ATCC, Rockville, MD, USA) and cultured in standard conditions".
Authentication	Cell lines were authenticated by genetic profiling using short tandem repeat analysis (AmpFISTR Identifiler PCR amplification kit, Thermo Fisher Scientific Inc, Waltham, MA, USA). This information is reported in the Section 'Cell lines and cell-based experiments' in the Methods.
Mycoplasma contamination	All cell lines tested negative for mycoplasma contamination, as assessed through MycoAlert® Mycoplasma Detection Kit (Lonza, Basel, Switzerland). This information is reported in the section 'Cell lines and cell-based experiments' in the Methods.

Commonly misidentified lines<br>(See ICLAC register)	None of the cell lines used in this study is listed in the database of commonly misidentified cell lines.
